# Community-based empowerment program to delay marriage: Results from the More Than Brides Alliance intervention in India, Malawi, Mali and Niger

**DOI:** 10.1371/journal.pone.0281413

**Published:** 2023-04-14

**Authors:** Andrea J. Melnikas, Grace Saul, Michelle Chau, Neelanajana Pandey, Mouhamadou Gueye, James Mkandawire, Aissa Diarra, Sajeda Amin

**Affiliations:** 1 Population Council, New York City, NY, United States of America; 2 Independent Consultant, New York City, NY, United States of America; 3 Population Council India, New Delhi, India; 4 Centre d’Études et de Recherche sur l’Information en Population et Santé, Bamako, Mali; 5 Invest in Knowledge Initiative, Zomba, Malawi; 6 Laboratoire d’Études et de Recherche sur les Dynamiques Sociales et le Développement Local, Niamey, Niger; IIPS: International Institute for Population Sciences, INDIA

## Abstract

The More Than Brides Alliance (MTBA) implemented an intervention in India, Malawi, Mali and Niger from 2017 to 2020. The holistic community-based program included girls’ clubs focused on empowerment and sexual and reproductive health knowledge; work with parents and educators; community edutainment events; and local-, regional-, and national-level advocacy efforts related to child marriage. Using a cluster randomized trial design (India and Malawi), and a matched comparison design (Niger and Mali), we evaluated the effectiveness of the program on age at marriage among girls ages 12–19 in intervention communities. Repeat cross sectional surveys were collected at baseline (2016/7), midline after approximately 18 months of intervention (2018), and endline (2020). Impact was assessed using difference-in-difference (DID) analysis, adjusted for the cluster design. We find that the intervention was successful at reducing the proportion of girls ages 12–19 married in India (-0.126, p < .001). Findings in the other countries did not show impact of the intervention on delaying marriage. Our findings suggest that the MTBA program was optimized to succeed in India, in part because it was built on an evidence base that relies heavily on data from South Asia. The drivers of child marriage in India may be substantially different from those in Malawi, Mali, and Niger and require alternate intervention approaches. These findings have implications for those designing programs outside of South Asia and suggest that programs need to consider context-specific drivers and whether and how evidence-based programs operate in relation to those drivers.

**Trial registration:** This work is part of an RCT registered August 4, 2016 in the AEA RCT registry identified as: AEAR CTR-0001463. See: https://www.socialscienceregistry.org/trials/1463.

## Introduction

Child marriage remains a significant human rights issue globally, with an estimated 12 million girls married before age 18 each year [1]. The consequences of child marriage are far-reaching and detrimental, including decreased educational attainment, increased health risks associated with early pregnancy and childbirth, higher incidence of intimate partner violence, and higher incidence of mental health problems [2–4]. While the causes of child marriage are context-specific, poverty and economic uncertainty are frequently cited as reasons for early marriage through pathways involving food insecurity, education expenses, or dowry costs associated with later age at marriage [5–7]. Evidence also suggests that premarital pregnancy and concerns about sexual security [8], as well as economic and social shocks related to climate change and displacement [6,9,10], natural disasters [11], or pandemics [12]—including the Covid-19 pandemic [13]—also threaten to exacerbate child marriage.

**Fig 1:**
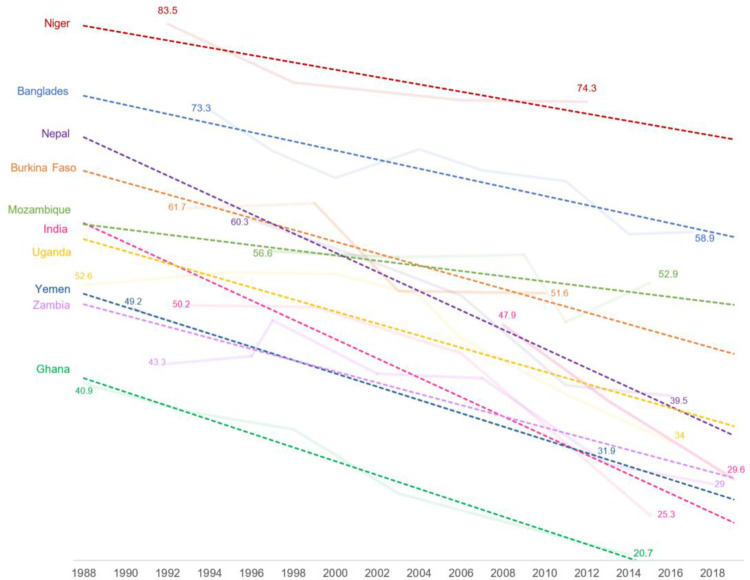
Trendlines in proportion of women ages 20–24 married by exact age 18, DHS 1988–2019. Bold, dotted lines show trendlines for each country. Light-shaded lines show available data, with earliest and latest datapoints represented for each country.

Over the past several decades, data show declines in child marriage globally—with the proportion of girls married by age 18 decreasing from 25% in 2008 to 21% in 2018 [14]—as well as across most impacted regions, however, there are significant discrepancies in trends between regions [15]. Fig 1 shows trendlines for the prevalence of marriage before age 18 (among women ages 20–24) from 1988 to present in UNICEF/UNFPA Global Programme to End Child Marriage countries where data are available prior to 2000. While the number of available datapoints is inconsistent across settings, trendlines show that while child marriage is declining everywhere, this is not occurring at the same rate across settings.

It is additionally important to note that even if countries experienced a similar rate of decline in the proportion of girls married as children, population size has a strong impact for determining a given country’s contribution to declines in child marriage worldwide. Much of the overall decline in child marriage is explained by a substantial drop in the rates of child marriage in a few large countries with high burdens of child marriage (i.e., a high absolute number of child brides), notably in India, which is home to one in three girls married before age 18 [15]. Over the past ten years, in South Asia, a girl’s risk of marrying before age 18 has declined by more than one third, from nearly 50% to 28% [15]. At the same time, some countries in other regions, such as Niger, Bangladesh, and Mali, the prevalence of child marriage remains stubbornly high. As global child marriage rates decline, the burden of child marriage is increasingly shifting to sub-Saharan Africa [15]. A recent report shows that child marriage declines have stagnated or that child marriage prevalence has even increased in some countries. In countries with high levels of institutional and social fragility, child marriage prevalence is nearly two times higher than the global average [15]. It is also notable that data showing large declines in child marriage prevalence in countries such as India care focused on national prevalence and in effect hide sub-national pockets where child marriage remains high [16].

A series of systematic reviews [17–20] suggest that empowerment programs and livelihoods approaches—including cash transfers to support girls’ education—appear to be the most promising approaches to address child marriage. It is notable, however, that these reviews represent a limited number of studies conducted in an even smaller number of countries. In the most recent systematic review of child marriage programs worldwide, for example, nearly half of included studies represented programs implemented in South Asia [20]. This bias toward South Asian contexts has important implications for the broader applicability of evidence-based strategies. In regions where the practice of child marriage is prevalent but has not been the subject of extensive intervention until recently—including in East and West Africa—there have been few studies examining program impact and even fewer assessing program appropriateness. Developing and implementing evidence-based programs in these settings is challenging when drivers of child marriage differ significantly from the contexts represented in the literature base.

This paper examines impact evaluation data from the More than Brides Alliance (MTBA) intervention in different contexts in India, Malawi, Mali, and Niger. We examine trends in marriage prevalence among girls ages 12–19 at the community level across settings characterized by either a high burden of child marriages (India) or a high proportion of child marriage (Malawi, Mali, Niger), and distinct drivers of the practice. According to the most recent Demographic and Health Surveys (DHS), the proportion of women ages 20–24 married before age 18 was approximately 76% in Niger (2012 DHS), 54% in Mali (2018 DHS), 42% in Malawi (2015–16 DHS), and 22% in India (2019–21 DHS).

We additionally examine program impact on other key outcomes for adolescent girls—including school enrollment, educational attainment, working experience, and knowledge about child marriage and sexual and reproductive health and rights (SRHR)—and consider what these results suggest about pathways to child marriage in the different program settings. This paper adds to the evidence base on whether strategies informed largely by evidence from the South Asian context translate to other contexts where child marriage is prevalent, including in West and Southeastern Africa.

## Intervention

**Fig 2:**
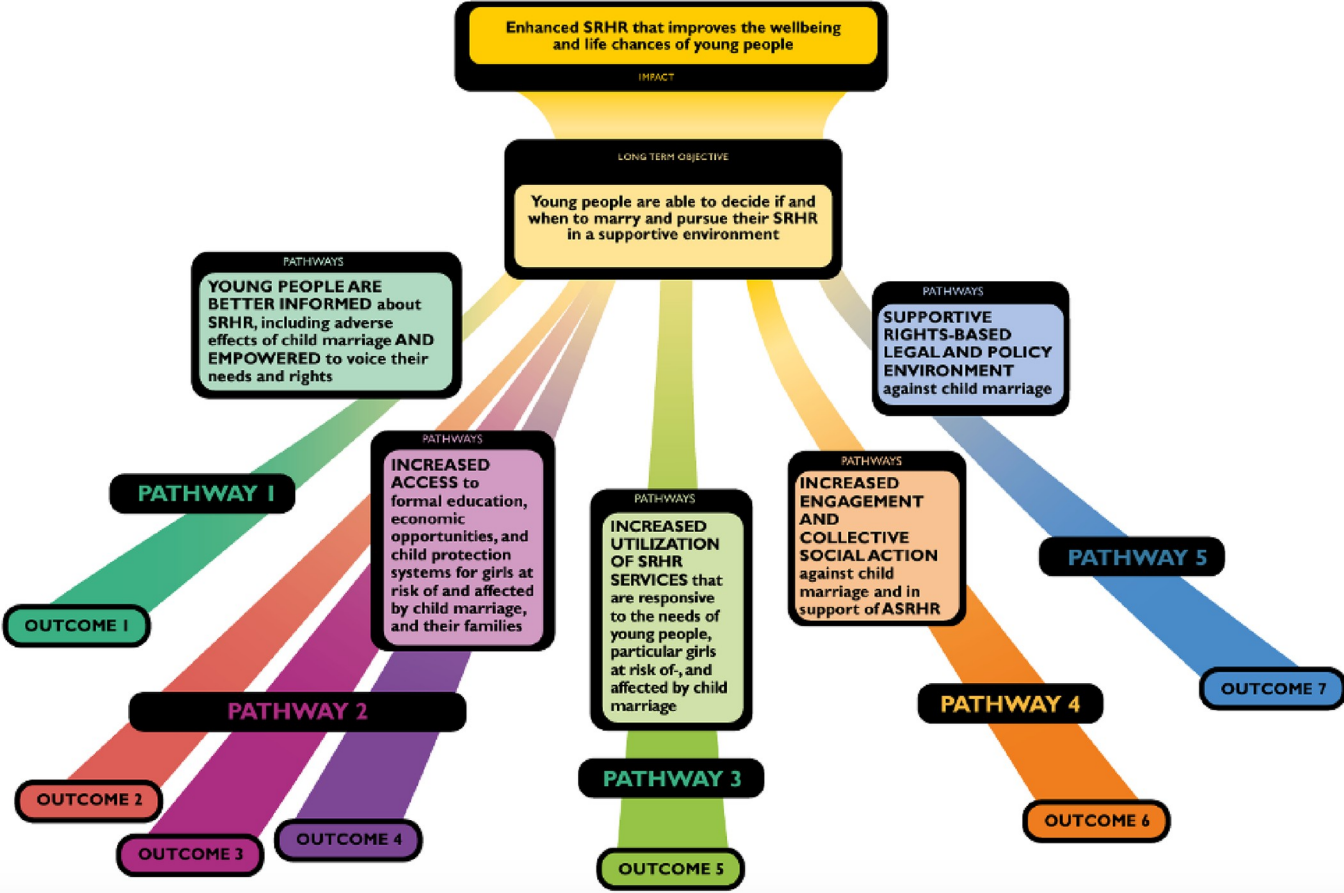
More Than Brides Alliance Theory of Change.

The MTBA intervention was designed in 2015 and sought to empower girls, to raise awareness about the risks of child marriage, to improve girls’ access to sexual and reproductive health (SRH) services, and to support social norms favorable to girls’ education, economic engagement, and agency in marital decision-making. The MTBA program approach included a package of interventions implemented at multiple levels and across sectors, including elements that were informed by the evidence base on child marriage interventions such as girls’ empowerment clubs and livelihood training for girls. According to the MTBA Theory of Change (ToC), reducing the prevalence of child marriage requires 1) **empowering girls** to make more informed decisions about marriage and their SRH, 2) **enhancing alternatives** to child marriage, and 3) creating an **enabling environment** in which girls can claim their rights. Fig 2 shows the MTBA program’s five pathways of change and their corresponding key outcome areas. For the purposes of program implementation, activities related to seven different outcome areas. In all countries, the intervention package put particular emphasis on Outcome 1 (“Young people are better informed about SRHR, including adverse effects of child marriage, and are empowered to voice their needs and rights”), and Outcome 6 (“Increased engagement and collective social action against child marriage in support of adolescent SRHR”).

The program was implemented by local community-based organizations from 2017–2020 in India, Malawi, Mali, Niger and Pakistan. In each country, an international program partner served as the lead organization responsible for project management and administration and for providing technical assistance to local implementing partners. Population Council served as the research partner in India, Mali, Malawi and Niger while Oxfam Novib conducted the research in Pakistan. Population Council India oversaw data collection in India while in Mali, Malawi and Niger, data were collected by local research organizations in partnership with Population Council. Our local research partners were the *Centre d’Études et de Recherche sur l’Information en Population et Santé* (CERIPS) in Mali, Invest in Knowledge Initiative (IKI) in Malawi, and the *Laboratoire d’Études et de Recherche sur les Dynamiques Sociales et le Développement Local* (LASDEL) in Niger.

## Methods

### Impact evaluation design

The evaluation followed a cluster randomized design in India and Malawi and a quasi-experimental (matched) design in Mali and Niger. A quasi-experimental design was used in Mali and Niger because the program aimed to build upon a previous intervention that had begun in 2015, so working in new (randomized) areas was not feasible. In Mali and Niger, we thus matched comparison villages to intervention villages on key criteria including population size, distance from the main road, and number of schools and health centers. We assessed balance at baseline (Table 1), and note that while the samples appear to be comparable in India and Malawi, in Mali and Niger, there were differences between intervention and comparison samples on several variables that may relate to key outcomes of interest. Analyses therefore adjust for these variables where possible.

**Table 1:** Baseline demographics across samples, by Country.

	MALI		MALAWI		NIGER		INDIA	
	Baseline		Baseline		Baseline		Baseline	
	Intervention	Comparison	Intervention	Comparison	Intervention	Comparison	Intervention	Comparison
Age (mean)	15.2	15.3	14.8	15	15.1	15.2	15.1	15
Ever married	11.9	16.9	16.2	15.1	25.7	37.7	19.8	18.2
Never attended school	32.7	40.8**	4.1	0.9***	29.7	46.5***	6.7	5.9
Not enrolled in school	33.1	38.5	35	31.4	27.5***	49	38.1	35.1*
Cannot read or write	44.3	55.4	32.3	21.4	45	64.0***	12.1	11.1
Ever pregnant (ages 15–19)	20	24.4	22.8	24.7	14.6	12.7	Not comparable (Asked among girls ever married and ages 15+)	

*** p < .01

**p < .05

*p < .10 difference between comparison and intervention.

**Fig 3:**
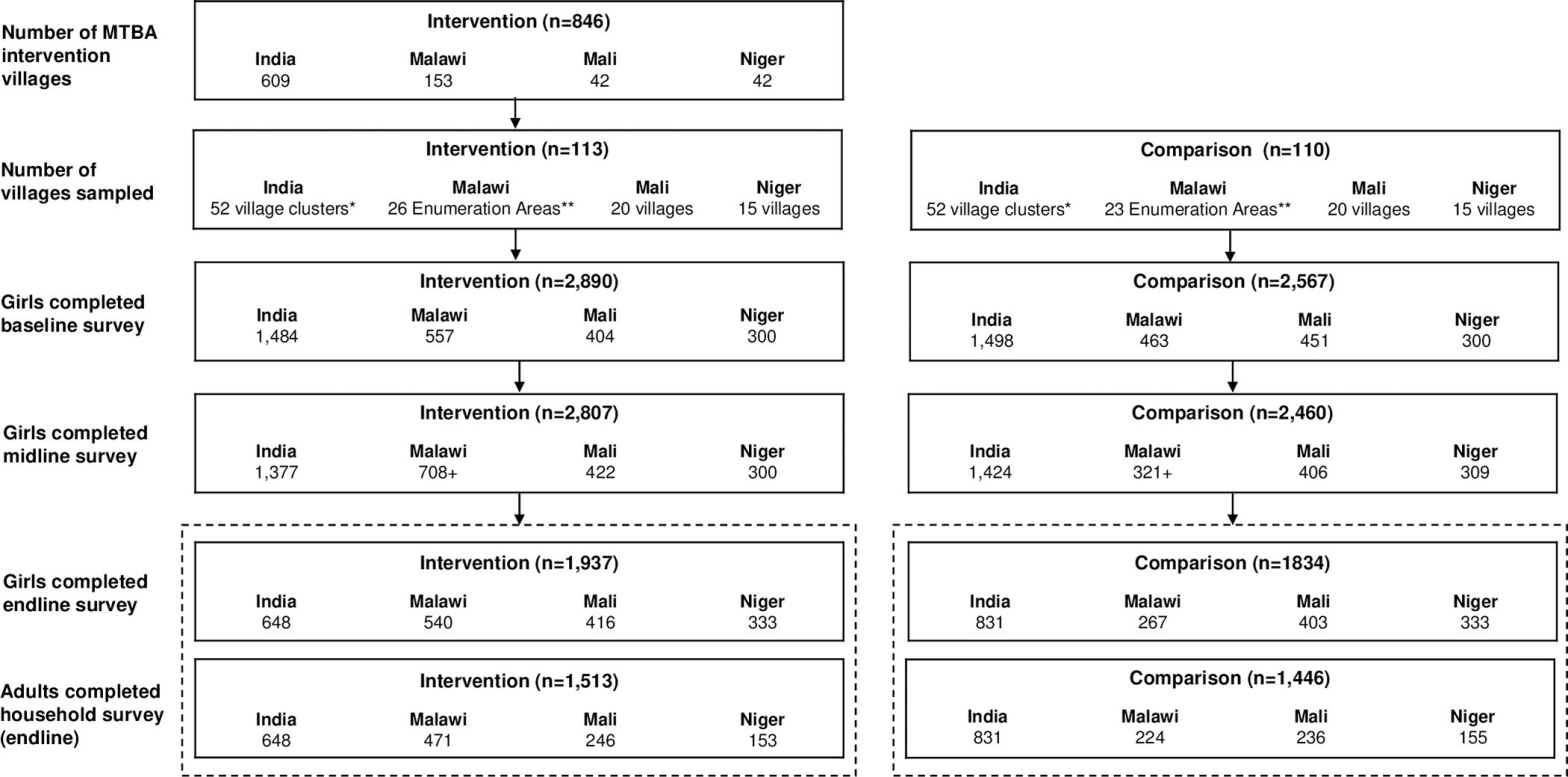
More Than Brides Alliance program and research samples. * If a village was smaller than 150 households, we created a village cluster grouping the small village to one or more neighboring villags within the same Panchayat. Village clusters were vetted by program partners. ** Enumeration Areas are geographic units defined by the Malawi National Statistical Office. We used this as the unit of measurement for sampling to maintain stability across rounds of data collection since the program used group head village (GHV) level, but those geographic units are subject to change. We mapped EAs and GHVs to "match" to each other and shared both EA and GHV assignments with program implementation staff. + Malawi comparison areas were reassigned at midline (from 26 intervention EAs to 31; from 23 comparison EAs to 14) based on contamination as the program was mistakenly implemented in planned comparison areas.

In all settings, we conducted repeated household listings followed by cross-sectional surveys with random samples of adolescent girls ages 12–19 drawn from intervention and comparison villages at baseline (2016/7), midline (2018) and endline (2020). At endline, to prevent the spread of Covid-19, we switched to remote data collection, conducting the adolescent girl surveys in two separate parts over the phone. Fig 3 shows sample sizes for adolescent girls 12–19 for each survey. We note overall slightly smaller sample sizes at endline due to the shift to remote data collection during the first year of the Covid-19 pandemic.

### Randomization and study participants

Clusters were randomized to either intervention or comparison areas (Malawi and India). In India, we stratified by states and then randomized clusters within each state. After completion of a household listing, girls ages 12–19 were randomly selected within clusters using a random number generator in Stata or Excel for inclusion in each survey (baseline, midline, endline). Enumerators interviewed randomly selected girls and contacted their households at three different times. If the girl was still not reachable after the enumerator’s third attempt, another respondent was randomly selected to replaced her in the sample.

In Mali and Niger, intervention villages had been previously selected by the program and thus randomization to intervention or comparison samples was not possible. We matched comparison villages to intervention villages on key criteria including population size, distance from the main road, and number of schools and health centers. We assessed comparability at baseline [23,24] and found the samples to be generally well balanced, except for on a few indicators, as noted above.

### Sample size and power analysis

We used the minimum detectable effect (MDE) approach to estimate sample sizes needed in each country. We used Optimal Design Software [21] for a multi-site cluster randomized trial (India and Malawi). Power was set at 0.80 and significance level at 0.05. The available fieldwork budget and program preferences for number of implementation sites led us to mutually agreeable total numbers of clusters and respondents per cluster, which varied from 50 clusters in India to 24 in Niger. Additional information on sampling is available elsewhere [22–25].

### Study instruments

Adolescent surveys were designed to capture key outcomes related to educational attainment, livelihood experience, gender-equitable attitudes, educational and marriage-related aspirations, marital status and history, basic literacy and numeracy skills, and knowledge of topics including marriage laws and SRHR. The endline instruments also included questions addressing the effects of Covid-19 and associated lockdowns on education, household finances, and marriage timing. Surveys were conducted by trained researchers using mobile phones or tablets and SurveyCTO online data collection tools. Surveys were conducted in local languages (Hindi, Odia, Hausa, Zarma, Bambara, Chichewe, Tonga, and Tumbuka) and enumerators could toggle between languages as needed. Data were downloaded from SurveyCTO daily and reviewed by research teams in each MTBA country and in the United States to ensure data quality.

### Outcomes

The main outcome of interest was proportion ‘ever married’, measured as the proportion of girls in the sample who reported ever having been married or living together with a partner as if married, including girls who were separated or divorced at the time of the survey. We also examined the proportion of girls ‘ever engaged’ and found that these results were similar to those for ever married. We therefore report ever married as the main outcome indicator. Other key outcomes in our analysis include ‘ever pregnant’, ‘currently enrolled in school’, and ‘ever worked for income’.

### Variation in samples

In Table 1 above, we show balance across intervention and comparison samples by country. In India and Malawi where areas were randomized to treatment, we see balance across most key demographic variables. In Mali and Niger—where areas were matched to the extent possible—we note that the proportion of girls who report never having attended school was significantly lower in comparison areas relative to intervention areas at baseline.

### Statistical analysis

Analyses were conducted in Stata SE 14.2 and included adjustments based on evaluation design and fidelity to randomization. We conducted difference-in-differences (DID) analyses using Stata SE 14.2 adjusted for the cluster design. In India and Malawi (where the intervention sites were randomized and balanced at baseline), we did not adjust for covariates. For India, we therefore present unadjusted results from the DID. In Malawi, we do not adjust for covariates but we do acknowledge that the program implementation deviated from the randomization slightly, with some intervention areas not receiving the program as intended and some comparison areas receiving the intervention. For Malawi, we therefore present the “as implemented” results, which more closely reflect the impact of the program. In Mali and Niger, the DID analysis included covariates, since the research design was quasi-experimental and the intervention and comparison samples differed on some key indicators at baseline. The DID analysis for Mali and Niger is adjusted for age, education level, wealth, and ethnicity. Age and education level were included at the individual level based on the adolescent girl survey responses. Household wealth and girls’ ethnicities were not collected in endline surveys. For this reason, wealth and ethnicity were calculated at the community level based on responses from adult endline surveys and adolescent surveys. Ethnicity was calculated as the proportion of the community belonging to the dominant ethnic group. Wealth was calculated as the average wealth score within the community. For the baseline and midline samples, wealth calculations were based on data from these respective survey years. For endline samples, wealth was calculated as an average of the baseline and midline responses within a given community.

### Ethics

Ethical and research clearance for this study was issued by the Institutional Review Board of the Population Council and by the National Committee on Research in the Social Sciences and Humanities (NCRSH) in Lilongwe (Malawi), the *Institut National de Recherche en Santé Publique* (INRSP) (Mali), and the *Comité d’Éthique pour la Recherche en Santé* (Niger). In India, data was collected by Population Council India, which is a trusted partner of the Government of India and adheres to the highest ethical standards while conducting clinical and non-clinical studies. For this study, the Population Council IRB reviewed and approved the protocol and deemed that obtaining additional local IRB for India was not necessary because all proposed research activities posed minimal risks to participants. The Population Council IRB follows the guidelines of the US Department of Health and Human Services under the Code of Federal Regulations.

Written informed consent was obtained from all research participants prior to their involvement in the study. For participants under the age of 18, written parental consent was obtained prior to contact with the adolescent participants, who were then asked to provide written asset if they wished to participate. For emancipated minors (married girls under age 18), parental consent was not required. At endline, consent was obtained from all participants orally, due to the shift of data collection to phone-based surveys. For minors, parents were contacted first and asked to provide oral consent for their children to be contacted and invited to participate in the study.

## Results

### Impact on child marriage

**Fig 4:**
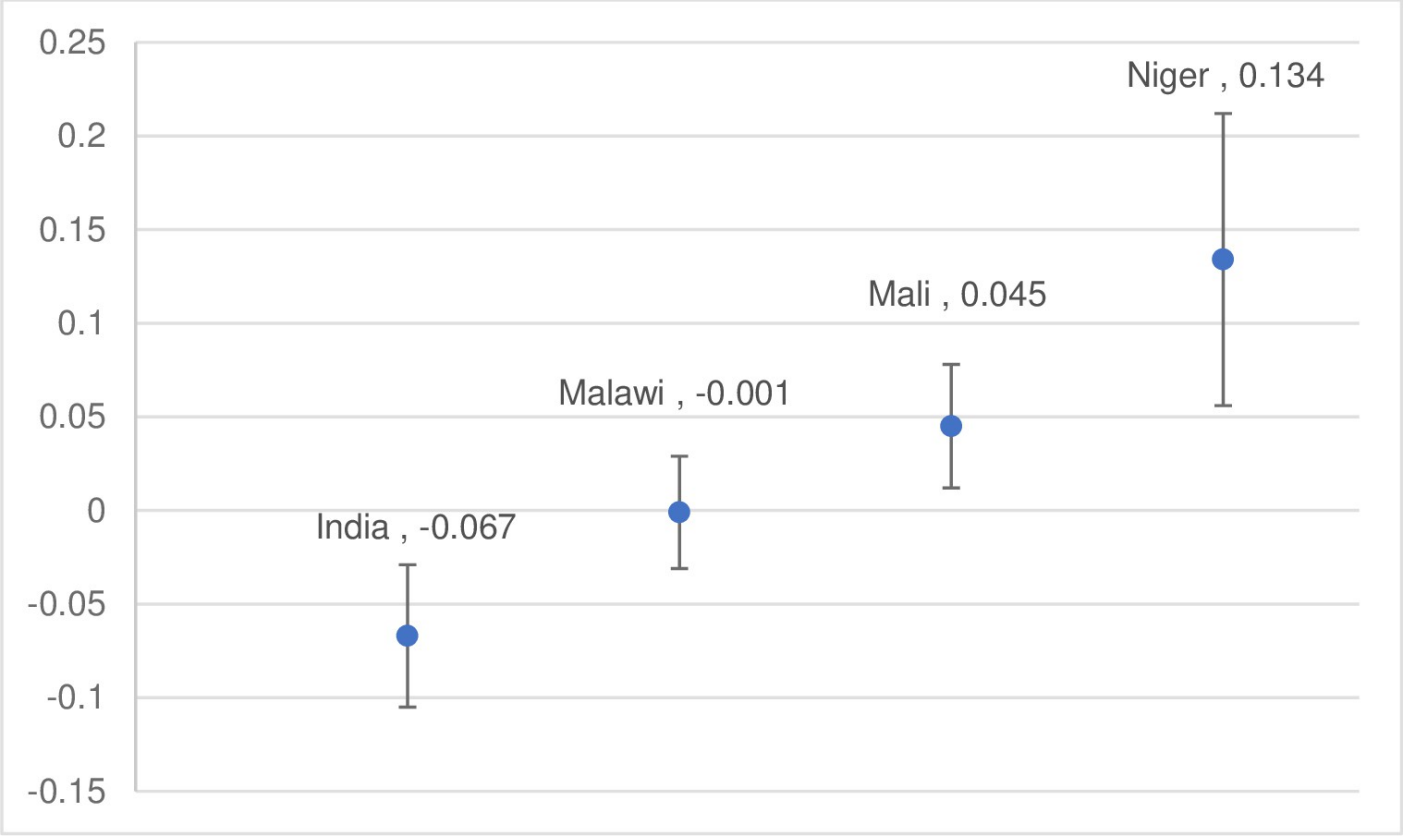
Difference-in difference results for proportion of girls ages 12–19 ever married, baseline to endline.

In Table 2, we present the proportion of girls ages 12–19 who reported ever being married at baseline and endline in intervention and comparison areas. We find that overall, the intervention was effective in reducing child marriage by endline in India, but not in the other three countries. In India, the proportion of girls married declined more in intervention areas than in comparison areas. Child marriage prevalence in intervention areas declined from 19.8% to 4.6%—a 77% decline overall—while in comparison areas, child marriage prevalence declined 47% (p < .05). Fig 4 presents DID analysis results. While the DID was significant in Niger, this was due to comparison areas experiencing a steeper decline in child marriage prevalence relative to intervention areas over the program period.

**Table 2:** Difference-in-differences, proportion of ever married girls ages 12–19.

	Intervention		Comparison		DID (se)	Sig (p)
	Baseline	Endline	Baseline	Endline		
India	19.8	4.6	18.2	9.6	-0.067 (0.026)	0.012**
**Malawi**	16.2	6.3	15.1	5.2	-0.001(0.030)	0.975
**Mali**	11.9	7.2	16.9	6.9	0.045 (0.033)	0.188
**Niger**	25.7	15.9	37.7	15.9	0.134(0.078)	0.098*

*** p < .01

**p < .05

*p < .10.

**Fig 5:**
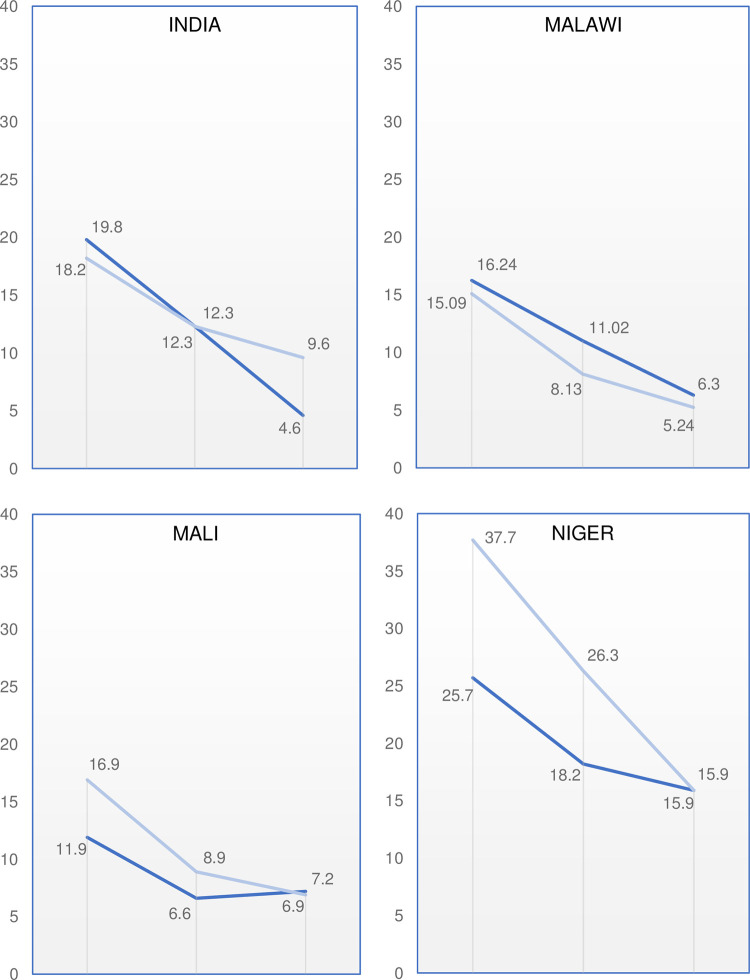
Changes in proportion of girls ever married at baseline, midline, and endline. Intervention areas shown in dark blue; comparisons in light blue.

In Fig 5, we show trends in child marriage prevalence in our samples since baseline, including midline data points. We see that child marriage was generally declining in all samples from baseline (2016/7) to midline (2018). In Malawi and Mali between baseline and midline, child marriage declined in intervention areas but also in comparison areas at a similar rate, and the DID was not significant. From midline to endline, child marriage prevalence continued to decline in intervention areas in India and Malawi. In Niger, the decline in child marriage prevalence observed in intervention villages from baseline to midline continued following midline but at a slower rate, while in Mali, the rate of child marriage decline plateaued from midline to endline (note: the 6.6% to 6.9% increase is not statistically significant).

### Impact on additional indicators

While the endline evaluation results did not find evidence that the MTBA intervention influenced child marriage prevalence in Malawi, Mali, or Niger, impact on other key indicators was detected. Table 3 gives the proportions observed at baseline and endline for all key indicators on which the DID analysis showed a significant difference between trends in intervention villages and comparison villages, suggesting that the MTBA intervention played a role in influencing the outcome. Full results of the evaluation can be found in the MTBA Endline Evaluation Report [26].

**Table 3:** More Than Brides Alliance intervention impact evaluation results.

		India		Malawi		Mali		Niger	
		BL	EL	BL	EL	BL	EL	BL	EL
**Marriage **									
Currently married	INT	14.8	4.5						
	COMP	12.4	9.6						
Mean age at marriage	INT					15.9	16.8		
	COMP					16.2	16.3		
Can correctly identify legal age at marriage	INT	61.9	88.6	44.5	59.6			15.0	29.6
	COMP	66.1	83.6	45.6	51.1			19.3	19.8
Can name at least three adverse effects of child marriage	INT	28.0	45.8					18.9	31.9
	COMP	26.4	34.8					40.7	17.9
**Health **									
Knows about HIV	INT	22.4	50.2						
	COMP	20.2	39.0						
Contraceptive knowledge scale (modern methods)	INT							48.3	64.4
	COMP							49.3	56.5
**Livelihoods **									
Currently working for income	INT							23.8	67.1
	COMP							66.0	72.5
**Education **									
Ever attended school	INT			95.9	97.8				
	COMP			99.0	98.0				
Currently enrolled in school (among ever enrolled)	INT	61.8	82.4						
	COMP	64.9	78.2						
Mean number of years of education completed	INT			3.9	4.5				
	COMP			4.7	4.6				
Cannot read or write	INT			32.3	14.4				
	COMP			21.4	13.2				
**Social life **									
Report being part of a club or group	INT	2.7	49.8						
	COMP	5.8	28.0						

In India, results show that the intervention increased the proportion of girls who were able to identify the legal age at marriage as well as the proportion of girls who were able to name at least three adverse effects of child marriage. The intervention additionally appeared to have increased knowledge of HIV, the proportion of girls enrolled in school, and the proportion of girls reporting being a member of a club or group. In Malawi, the intervention appears to have increased the proportion of girls who were able to correctly identify the legal age at marriage for girls and improved outcomes on multiple education-related indicators, namely the proportion of girls who had ever attended school, the mean number of years of education completed, and the proportion of girls who self-reported the ability to read or write. In Mali, the only significant difference between the changes observed in intervention and comparison communities between baseline and endline was an increase in the mean age at marriage in intervention communities. Lastly, in Niger, the intervention appears to have influenced two key indicators related to child marriage: the proportion of girls able to correctly identify the legal age at marriage for girls, and the proportion of girls able to name at least three adverse effects of child marriage. The intervention also appears to have improved girls’ knowledge about modern contraceptive methods and increased the proportion of girls currently working for income.

## Discussion

The MTBA impact evaluation offered an important opportunity to understand how a program with the same basic design performs across four contexts characterized by divergent drivers of child marriage. As the results show, at the end of the MTBA intervention, the evaluation was able to detect program impact on child marriage prevalence in India, but not in Malawi, Mali, or Niger. In intervention areas in India, the observed declines in child marriage from baseline to midline were maintained from midline to endline while in comparison areas, child marriage prevalence declined from baseline to midline and slowed from midline to endline. Although data from the other three MTBA countries showed encouraging declines in child marriage from baseline to endline, we are unable to attribute these trends to the MTBA program, indicating that observed declines may be the result of broader trends toward delayed marriage for adolescent girls occurring in these settings.

Reflecting on this primary result of impact on child marriage prevalence in India but not elsewhere, we note that the MTBA program was designed to build upon the body of evidence on what program approaches work to reduce child marriage that was available during the program’s inception period in 2016 [17,18]. Since relatively few studies meet the criteria for inclusion in systematic reviews, the evidence base does not fully capture the impact of child marriage interventions in different contexts and thus there may be important limitations in the applicability of current evidence-based recommendations across settings. The most comprehensive systematic review of child marriage program evaluations to-date [20], for example, includes just 30 studies, 14 of which relate to programs implemented in South Asia. As child marriage programming has a longer history in South Asian countries and programmatic strategies are more deeply entrenched in the region, the strategies that are documented in the evidence base are likely best adapted to target drivers of child marriage in South Asian contexts. Assumptions that engaging girls’ parents as key decision-makers in the marriage formation process or that working to “empower” girls to raise their voices in favor of their rights and to reject early marriage will yield results, for example, do not appear to hold in all contexts (particularly in settings where arranged marriage is not the norm). We hypothesize that particular context-specific drivers that are underrepresented in the evidence base help to explain the differential results of the MTBA intervention on child marriage prevalence observed across countries.

In Niger, the program successfully increased girls’ knowledge of the legal age of marriage for girls (15 years), of the adverse effects of child marriage, and of modern contraceptive methods at the community level, however, these increases in knowledge did not appear to translate into behavior change with respect to age at marriage. Qualitative research in MTBA intervention villages in Niger has demonstrated that girls themselves often express preference for early marriage, viewing marriage as the most desirable pathway and as a rite of passage to adulthood, bringing with it the admiration of their peers, the respect of others in the community, and increased independence from their parents [27]. In this context, parents and girls alike reported that girls themselves play an important role in the marriage formation process and that girls are not forced to marry against their will [27]. These findings complicate notions of “agency” and “empowerment” as typically presented within child marriage programming. In Niger, girls’ attitudes toward marriage, the absence of desirable educational or economic opportunities available to girls, and a lack of female role models who have chosen to pursue an alternative pathway are important contextual factors to target in child marriage programming [27]. The MTBA intervention’s impact on increasing the proportion of girls who reported currently working for money, however, appears to be a positive and potential intermediary result that may lead to declining child marriage rates in time, if girls’ increasing involvement in income-earning activities influences girls’ perceptions of the opportunities available to them and the potential benefits of delaying marriage.

Similarly, in Malawi, we found that the assumption that increased awareness of age at marriage laws and girls’ rights would lead to norms change and reduced prevalence of child marriage does not appear to hold. Through qualitative research in Malawi, we found that while knowledge of laws is high, punitive responses emphasizing enforcement of minimum age at marriage laws do not appear to influence marriage timing [28], as they fail to address important proximal causes of child marriage in Malawi, namely premarital pregnancy [29]. We hypothesize that a main reason the MTBA intervention failed to impact child marriage in Malawi is because it did not succeed in reducing the prevalence of premarital pregnancy. While the intervention did appear to improve several key indicators related to education, no impact was found on the prevalence of premarital pregnancy. Reactive strategies—namely, the practice of “marriage withdrawal” whereby married girls are removed from their marital homes, returned to their natal homes, and re-enrolled in school—may improve indicators related to education, but will not influence the proportion of girls *ever* married in the community. Qualitative research examining marriage withdrawal also indicates that girls withdrawn from marriages have often already begun childbearing and may face stigma and limited educational and livelihood opportunities following the dissolution of their marriages [28].

In Mali, the assumption that a community-based intervention could impact child marriage prevalence did not consider the reality of highly mobile populations in this context. In some areas where the program operated in Mali—especially in rural Ségou—temporary migration is common among adolescent girls, often for the purpose of earning money to build a “trousseau” (an accumulation of personal and household items collected in preparation for marriage) [30]. Temporary migration for schooling and seasonal agricultural labor is also common. Where adolescent migration rates are high, repeat cross-sectional surveys will result in an undercounting of girls who are attending school, are engaged in income-generating activities, or are married, if girls have moved away for one of these reasons. At the time of the MTBA midline household listing, CERIPS data collectors signaled a high number of girls absent from their villages, prompting the addition of a question about temporary migration to be added to the household listing tool. In the remaining villages listed, we found that 18% of girls eligible for inclusion in the survey were absent from their villages, indicating a high level of mobility [25]. Absent girls who were randomly selected for inclusion in the study had to be replaced by girls who were present at the time of the surveys, which may have obscured the true impact of the intervention, particularly if girls who were absent from their communities had been engaged with the program previously. We posit that the lack of program impact on child marriage and other key indicators detected in Mali is due to the compositional instability of the population included in the evaluation

### Limitations

There are a few key limitations to note. First, we acknowledge that there was a difference in research design in India and Malawi (cluster randomized cross sectional design) versus Mali and Niger (quasi experimental matched design). As such, the lack of results in Mali and Niger may be due in part to the challenges of finding suitable comparison communities and be explained by contextual differences between those countries and India. Niger samples sizes may also be too limited to detect change; while power calculations used proportion of girls ages 15–19 married using DHS data from 2012, the proportion of girls found to be ever married at baseline was substantially lower than expected. While our focus was on measuring impact on key outcomes for girls at the community level, we acknowledge that including only adolescent girls in the sample limited our ability to make inferences about changes in community norms more broadly. Additionally, we note that child marriage is declining everywhere (Fig 1) and that our results show a similar trend. The data at endline were also collected remotely due to the 2020 Covid-19 pandemic. While we address the change in data collection mode in analyses, the Covid-19 pandemic may have had effects on outcomes that are not visible in our data. In India, we see that in intervention areas, child marriage continued to decline even during the Covid-19 pandemic, while in comparison areas, it the rate of decline slowed, plateaued, or even increased [31].

Despite these limitations, we believe our results provide important information about the context-specific questions that arise in understanding the impact of community-focused asset-building programs to address child marriage. Our findings suggest that these programs can still be highly effective in contexts that share similarities with the evidence base built on programs in South Asia, including areas where parents are often involved in decisions about girls’ marriages, alternative education and livelihood options exist for girls and women, and other drivers of child marriage—such as migration or premarital sex—are less influential. The lack of demonstrated effectiveness of the MTBA intervention in Niger, Malawi and Mali suggests that programs in these contexts need to be better tailored to specific drivers of child marriage in those settings, as noted in Psaki et al 2021 [32]. Our findings also support a need for better evidence on successful programmatic approaches, particularly in West Africa where child marriage prevalence remains stubbornly high [33] and climate change and instability threaten to exacerbate the practice [34].

## Conclusion

Child marriage is declining everywhere, but there are notable discrepancies in the changes observed between contexts: much of the change is driven by declines in countries where drivers of child marriage have been widely documented and where child marriage programs have a deeper history of engagement. On the question of what works to delay marriage for girls in countries with high rates of child marriage, it is important to recognize that we don’t have the same standard of evidence in contexts beyond South Asia. Evidence-based programming approaches are therefore likely best adapted to drivers that are more prominent in South Asian contexts.

While child marriage prevalence remains deeply entrenched in many contexts, there is a tendency for research and programming efforts to operate in settings where child marriage is already declining. As recognition of the importance of tailoring program strategies to context-specific drivers of child marriage increases, there is a great demand for rigorous research to explore how various drivers interact in specific settings and to measure the impact of program strategies being implemented.
